# Two new psathyrelloid species of *Coprinopsis* (Agaricales, Psathyrellaceae) from China

**DOI:** 10.3897/mycokeys.83.71405

**Published:** 2021-09-08

**Authors:** Gu Rao, Dan Dai, Hui-Nan Zhao, Yi Liang, Yu Li, Bo Zhang

**Affiliations:** 1 Engineering Research Center of Edible and Medicinal Fungi, Ministry of Education, Jilin Agricultural University, Changchun, Jilin 130118, Changchun, China Jilin Agricultural University Changchun China

**Keywords:** Asia, molecular systematics, morphology, new taxa, taxonomy

## Abstract

In this study, *Coprinopsisjilinensis* and *Coprinopsispusilla* were introduced, based on their morphological characteristics, the internal transcribed spacer (ITS) and large subunit ribosomal (LSU) region sequences of nrDNA. These new psathyrelloid species were found in Jilin Province, China. *Coprinopsisjilinensis* has brown pileus, utriform pleurocystidia, brown, smooth, dextrinoid basidiospores and tiny pore. It mainly grows on humus. *Coprinopsispusilla* has small basidiomata, greyish-white pileus, thick and distinct veil at edges, subcolourless and verrucose basidiospores. It is poreless and it grows on the decaying wood of broad-leaved trees. Both of them belong to the C.sect.Melanthinae. A supplementary description of C.sect.Melanthinae was given in combination with the newly-discovered taxa and an identification key to the fourteen psathyrelloid species of *Coprinopsis* is provided. Coprinopsissect.Canocipes and C.sect.Quartoconatae were evaluated and the phylogenetic position of the psathyrelloid species of *Coprinopsis* was discussed. *Psathyrellasubagraria*, as a confusing species, was also discussed in this study.

## Introduction

Coprinoid mushrooms are fascinating fungal taxa with the characteristic of deliquescent lamellae. *Coprinus* sensu lato is not monophyletic (Johnson and Vilgalys 1998; Hopple and Vilgalys 1999; Park et al. 1999a, 1999b; Moncalvo et al. 2002). Based on molecular studies, Redhead et al. (2001) subdivided *Coprinus* s. l. into four genera, *Coprinus* Pers. (Agaricaceae), *Corinopsis* P. Karst. (Psathyrellaceae), *Coprinellus* P. Karst. (Psathyrellaceae) and *Parasola* Redhead, Vilgalys & Hopple (Psathyrellaceae). Combined with anatomical characteristics, 100 species were divided from *Coprinus* s. l. into *Coprinopsis*, which were then widely accepted. The subdivision of the *Coprinus* s. l. was full of controversies at the time. Noordeloos et al. (2005) argued that there were many unresolved problems in the phylogeny and it was too early to subdivide it into four genera.

Schafer (2010) used the classification system of Redhead et al. (2001) to divide *Coprinopsis* into five sections, *Atramentarii*, *Lanatuli*, *Alachuani*, *Narcotici* and *Nivei*, corresponding to Coprinussect.Atramentarii (Fries 1836), C.sect.Lanatuli (Fries 1836), *C.C.* subsect. *Alachuani* (Singer 1949), *C.C.* subsect. *Narcotici* (Uljé and Noordeloos 1993) and *C.C.* subsect. *Nivei* (Citerin 1992), but the traditional section C.sect.Picacei (Kauffman 1918) was not covered. Wächter and Melzer (2020) subdivided the genus *Coprinopsis* into 20 sections according to the subclades of the phylogenetic tree (*Cinereae*, *Filamentiferae*, *Melanthinae*, *Alopeciae*, *Xenobiae*, *Phlyctidosporae*, *Krieglsteinerorum*, *Erythrocephalae*, *Geesteranorum*, *Mitraesporae*, *Radiatae*, *Subniveae* and *Canocipes*).

*Coprinopsis* is a worldwide fungal taxon that includes some well-known species, such as *C.atramentaria*, characterised by hyphal pileus cuticle, abundant powdery to floccose veil covering the whole pileus, coprophilous, growing in a terrestrial or lignicolous habitat (Redhead et al. 2001). According to Kirk et al. (2008), 200 species of *Coprinopsis* were known so far the world over. At the time of submission, there are 228 records of *Coprinopsis* in Index Fungorum (www.indexfungorum.org), including synonyms, varieties, forms and names. There are few studies on *Coprinopsis* in China. According to the report of Huang (2019), there were only 16 species of *Coprinopsis* in China, amongst which eight species were newly recorded in China and reported in 2019. Based on traditional morphology, sequence data and phylogenetic analyses, two new species of *Coprinopsis* were found in Jilin Province, China. They both belong to the C.sect.Melanthinae and will be reported as follows.

## Materials and methods

### Collecting and morphological studies

The fresh basidiomata were collected from the Red Leaves Valley in Hanchongling (approximate 43°02'1.67"N, 127°59'36.55"E), Dunhua City, Yanbian Korean Autonomous Prefecture, Jilin Province, China. After dried at 45 °C for 1‒2 days, they were stored in the Herbarium of Mycology of Jilin Agricultural University (HMJAU). Photos of fresh basidiomata were taken in the field. The macromorphology was observed from fresh basidiomata and the observation of microstructure was based on dry specimens under a light microscope (LEICA DM1000). The mainly used reagents are 5% potassium hydroxide (KOH) solution, 1% Congo Red and Melzer’s Reagent. The morphological description referred to Largent et al. (1977 and 1978). The surface of the basidiospores was observed and photographed under a scanning electron microscope (SEM) (Hitachi SU8000) at 2.0 kV, with a working distance of 8 mm. The following symbols were used in the description: [n/m/p] indicates that ‘n’ randomly selected basidiospores from ‘m’ basidiomata of ‘p’ collections were measured, ‘avl’ means the average length of basidiospores, except the extreme values, ‘avw’ means the average width of the basidiospores, except the extreme values, ‘Q’ represents the quotient of the length and width of a single basidiospores in side view, ‘Q_m_’ refers to the average Q value of all basidiospores ± standard deviation. Dimensions for basidiospores are given as (a) b–c (d). The range of b–c contains a minimum of 90% of the measured values. Extreme values (i.e. a and b) are given in parentheses.

### Research methods of molecular systematics

The total DNA of the specimens was extracted by the new plant genomic DNA extraction kit from Jiangsu Kangwei Century Biotechnology Company Limited. The amplification primers of LSU nrDNA (LSU) were LROR and LR5 (Vilgalys and Hester 1990), the ITS nrDNA (ITS) regions were ITS1 and ITS4 (White et al. 1990; Gardes and Bruns 1993). The amplification reactions were carried out in a 25 μl system and the total amount of the reactions was as follows: ddH_2_O 13.5 μl, 10×Taq Buffer 5 μl, 10 mM dNTPs 1 μl, 10 mM upstream primer 1 μl, 10 mM downstream primer 1 μl, DNA sample 2 μl, 2 U/μm Taq Polymerase 1.5 μl. The cycle parameters were as follows: 4 min at 94 °C for 1 cycle; 40 s at 94 °C, 40 s at 54 °C, 1 min at 72 °C for 35 cycles; 10 min at 72 °C for 1 cycle; storage at 4 °C. The PCR product was subjected to 0.5% agarose gel electrophoresis to test strips. The sequencing work was entrusted to Shenggong Bioengineering (Shanghai) Company Limited and the sequencing results were clipped with Seqman 7.1.0 (Swindell and Plasterer 1997) and then submitted to GenBank (https://www.ncbi.nlm.nih.gov/genbank/). The newly-obtained sequences are shown in Table 1. The sequences of relevant taxa were downloaded from GenBank and from the related articles (Larsson and Örstadius 2008; Nagy et al. 2010; Nagy 2012; Örstadius et al. 2015; Crous 2017; Melzer et al. 2017).

**Table 1.** Taxa, vouchers and sequence accession numbers of newly generated sequences.

Taxon	Voucher	ITS nrDNA	LSU nrDNA
* Coprinopsis pusilla *	HMJAU 58779	MZ398012	MZ398067
* C. pusilla *	HMJAU 58780	MZ398013	MZ398068
* C. pusilla *	HMJAU 58781	MZ398014	MZ398069
* C. jilinensis *	HMJAU 58782	MZ398015	MZ398070
* C. jilinensis *	HMJAU 58783	MZ398016	MZ398071

The ‘auto’ strategy and normal alignment mode of MAFFT (Katoh et al. 2005) were used for Sequence alignment and Gblocks (Castresana et al. 2000; Talavera and Castresana 2007) was used to obtain the conservative segments of sequences with the following parameters: the minimum number of sequences for a conserved/flank position (12/12), the maximum number of contiguous non-conserved positions (8), minimum length of a block (10) and allowed gap positions (with half). ModelFinder (Kalyaanamoorthy et al. 2017) was used to select the best-fit models using the Bayesian Information Criterion (BIC). The Maximum Likelihood (ML) analyses were performed in IQTree 1.6.8 (Nguyen et al. 2015) and the Bayesian Inference phylogenies were performed in MrBayes 3.2.6 (Ronquist et al. 2012) (2 parallel runs, 2000000 generations), in which the initial 25% of sampled data were discarded as burn-in. The above software was integrated into PhyloSuite 1.2.2 (Zhang et al. 2020). The neighbour-joining (NJ) tree was carried out in Mega X (Kumar et al. 2018). The ML and NJ trees were evaluated by bootstrap analysis with 1000 replicates and the best models are shown in Table 2.

**Table 2.** The best models, based on ModelFinder and MegaX.

	BI	ML	NJ
ITS nrDNA	SYM+G4	TVMe+G4	T92+G5
LSU nrDNA	K2P+I	TIM2+F+I	K2+G5

## Results

### BLASTn results

In the BLASTn alignment, based on ITS sequences, *Coprinopsispusilla* and *C.melanthina*KC992961 (Örstadius et al. 2015) had the highest sequence identities (94.92%–95.26%), with 34–36 base differences. *Corpinopsisjilinensis* and *C.uliginicola*MG712323 (Zhang 2019) had the highest sequence identity (99.27%), with five base differences. The sequence identities between *C.jilinensis* and *C.uliginicola*KC992960 Type (Örstadius et al. 2015) were 93.98%–94.35%, with 39–42 base differences. In BLASTn alignment, based on LSU sequences, *C.pusilla* and *C.melanthina*KC992961 (Örstadius et al. 2015) had the highest sequence identities (99.14%–99.25%), with 7–8 base differences. *Corpinopsisjilinensis* and *C.uliginicola*KC992960 Type (Örstadius et al. 2015) had the highest sequence identity (99.13%), with eight base differences.

### Phylogenetic analyses

**Figure 1.**
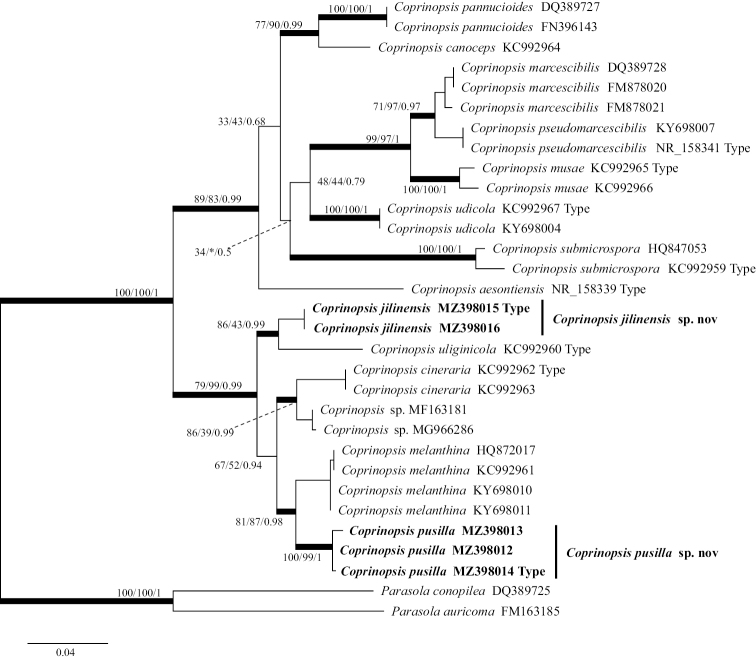
The phylogenetic tree of *Coprinopsis* by ITS nrDNA, based on the Maximum Likelihood method (ML). The three values of internal nodes respectively represent Maximum Likelihood bootstrap (MLBP)/neighbour-joining bootstrap (NJBP)/Bayesian posterior probability (BIPP). The thick node indicates the signiﬁcantly-supported branch in at least two analyses (MLBP ≥ 70, NJBP ≥ 70, BIPP ≥ 95%). The GenBank accession number is marked after the species name. At the same time, the sequence from the type specimen is also marked at the end. Two new species from China are expressed in bold font and *Parasolaconopilea* (Fr.) Örstadius & E. Larss and *P.auricoma* (Pat.) Redhead, Vilgalys & Hopple are selected as the outgroups.

**Figure 2.**
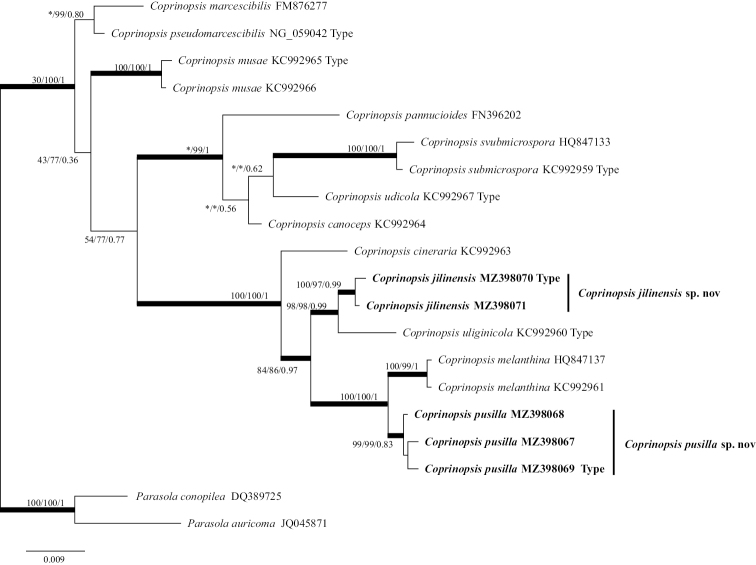
The phylogenetic tree of *Coprinopsis* by LSU nrDNA, based on Bayesian Inference (BI). The three values of internal nodes respectively represent Maximum Likelihood bootstrap (MLBP)/neighbour-joining bootstrap (NJBP)/Bayesian posterior probability (BIPP). The thick node indicates the signiﬁcantly-supported branch in at least two analyses (MLBP ≥ 70, NJBP ≥ 70, BIPP ≥ 95%). The GenBank accession number is marked after the species name. At the same time, the sequence from the type specimen is also marked at the end. Two new species from China are expressed in bold font and *Parasolaconopilea* (Fr.) Örstadius & E. Larss and *P.auricoma* (Pat.) Redhead, Vilgalys & Hopple are selected as the outgroups.

After Gblocks clipping, the ITS data matrix included 31 sequences of 588 nucleotide sites from 16 taxa and the data matrix included 20 sequences of 921 nucleotide sites from 14 taxa (gaps included). In the ITS and LSU phylogenetic trees (Figs 1, 2), *Coprinopsispusilla* and *C.jilinensis* both belong to C.sect.Melanthinae. *Coprinopsispusilla* and *C.melanthina* formed a sister clade, *C.jilinensis* and *C.uliginicola* formed a sister clade, both of which were strongly supported. In the phylogenetic trees, based on ITS sequences (Fig. 1), *C.submicrospora* and *C.marcescibilis*/*C.pseudomarcescibilis*/*C.musae*/*C.udicola* formed a sister clade in the ML and BI trees (MLBP/BIPP: 34/0.5), but *C.submicrospora* and *C.canoceps*/*C.pannucioides* formed a sister clade in the NJ tree (NJBP: 39). In the phylogenetic trees, based on LSU nrDNA sequences (Fig. 2), *C.marcescibilis* and *C.pseudomarcescibilis* formed a sister clade with strong support in the NJ and BI trees (NJBP/BIPP: 99/0.8). However, in the ML tree, *C.marcescibilis* formed a sister clade with all taxa, except the outgroups (MLBP: 100) and *C.pseudomarcescibilis* formed a sister clade with all taxa, except the outgroups and *C.marcescibilis* (MLBP: 30). In the analyses, based on different methods, the sister branch relationships of *C.pannucioides*, *C.submicrospora*, *C.uliginicola* and *C.canoceps* were different. In the BI tree, *C.pannucioides* and *C.submicrospora*/*C.udicola*/*C.canoceps* formed a sister clade with strong support (BIPP: 1), *C.canoceps* and *C.submicrospora*/*C.udicola* formed a sister clade (BIPP: 0.56), *C.submicrospora* and *C.udicola* formed a sister clade (BIPP: 0.62). In the ML tree, *C.submicrospora* and *C.udicola*/*C.canoceps*/*C.pannucioides* formed a sister clade with strong support (MLBP: 98), *C.udicola* formed a sister clade with *C.pannucioides*/*C.canoceps* (MLBP: 30) and *C.pannucioides* and *C.canoceps* formed a sister clade (MLBP: 50). In the NJ tree, *C.pannucioides* and *C.submicrospora*/*C.udicola*/*C.canoceps* formed a sister clade with strong support (NJBP: 99), *C.submicrospora* formed a sister clade with *C.udicola* /*C.canoceps* (NJBP: 38) and *C.udicola* and *C.canoceps* formed a sister clade (NJBP: 41).

### Taxonomy

#### Coprinopsis jilinensis

Taxon classificationFungiAgaricalesPsathyrellaceae

G. Rao, H.N. Zhao, B. Zhang & Y. Li sp. nov.

520AEB58-852A-5349-826B-5C97221239E7

840297

**Figure 3.**
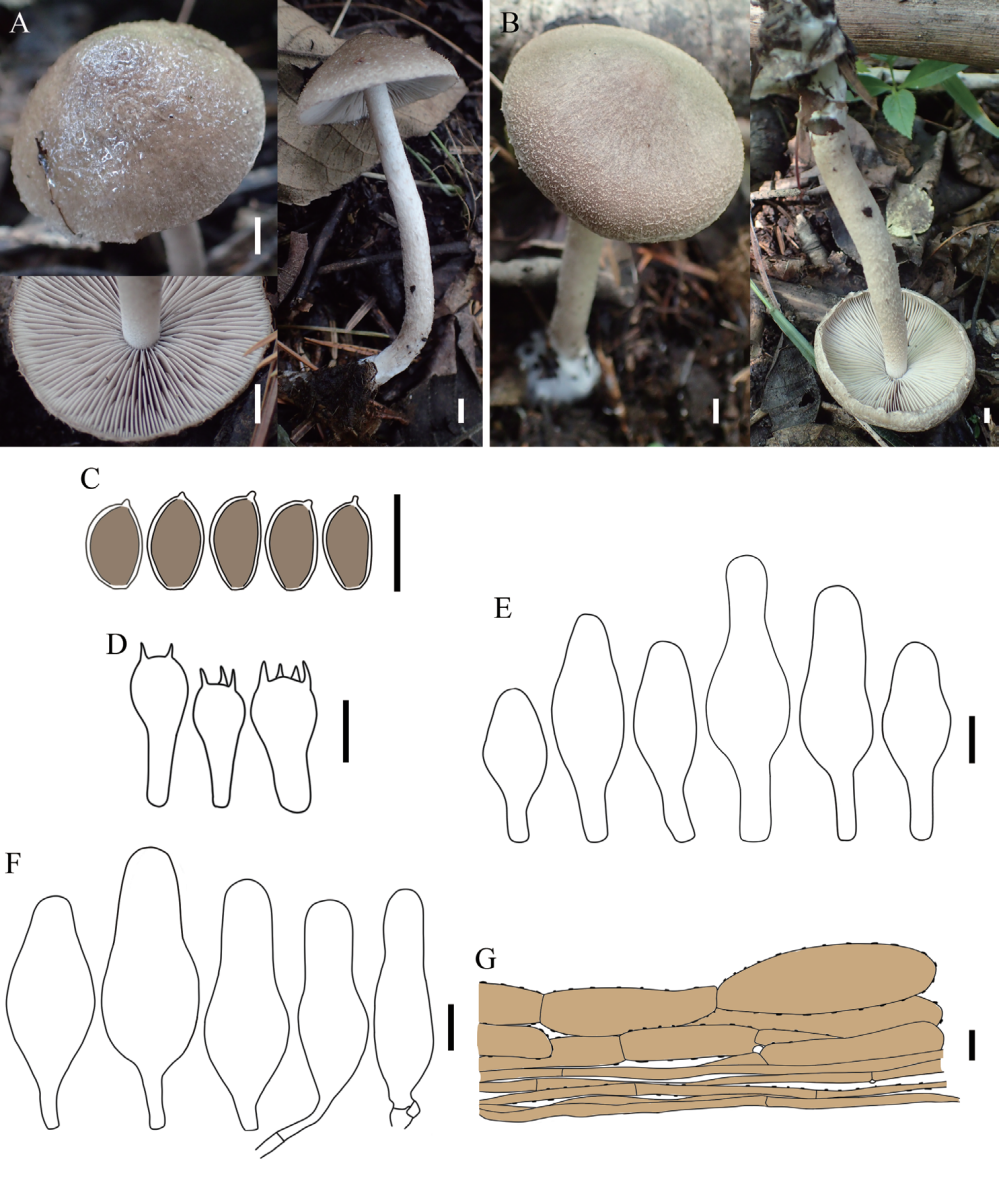
Basidiomata and microscopic features of *Coprinopsisjilinensis***A** collection HMJAU 58783 **B** collection HMJAU 58782 **C** basidiospores **D** basidia **E** pleurocystidia **F** Cheilocystidia **G** pileipellis. Scale bars: 5 mm (**A, B**); 10 µm (**C–G**).

**Figure 4.**
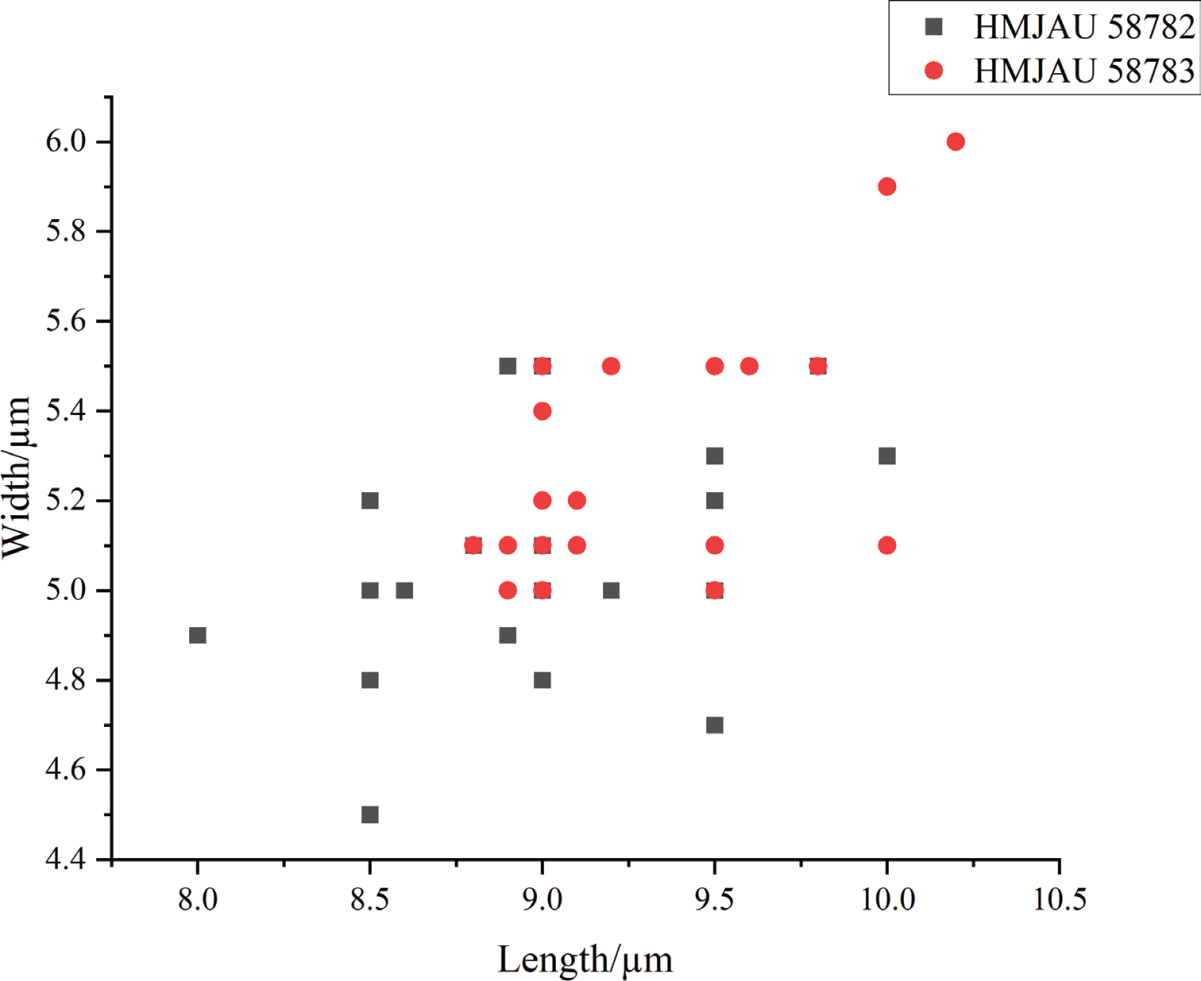
Scatter plot of basidiospores size in *Coprinopsisjilinensis*.

Figures 3 , 4 , 7C, D

##### Typification.

China. Red Leaves Valley in Hanchongling, Dunhua City, Yanbian Korean Autonomous Prefecture, Jilin Province, 22 August 2019, G. Rao & H.N Zhao (HMJAU 58782 Holotype!).

##### Sequences ex holotype.

MZ398015 (ITS nrDNA), MZ398070 (LSU nrDNA).

##### Etymology.

The epithet “*jilinensis*” refers to this species that was first discovered in Jilin Province, China.

##### Description.

Basidiomata small to medium-sized. Pileus 33–52 mm broad, conical to convex, dark brown or clay brown, densely covered with white hairs, not sticky when dry or wet, not hygrophanous, veil remnants flocculent at edges. Lamellae close or crowded, grey-white to fleshy brown, brownish-black after drying, sinuate or adnexed, not the same length and width, edges slightly toothed, concolorous, not deliquescent. Stipe 80–95 × 5–9 mm, white to milky white, cylindrical, down slightly rough, fibrous, a little fragile, hollow, the base with white mycelium, dense or sparse, close to the stipe surface covered with brownish-yellow pubescent, no ring. Spore print without record.

Basidiospores [60, 2, 2] (8)8.5–10(10.2) × 4.5–5.9 (6) µm, avl = 9.1 µm, avw = 5.2 µm, Q = (1.62) 1.63–1.96 (2.02), Q_m_ = 1.77 ± 0.09, oval to long oval, brown, brownish-yellow or dark brown in 5% KOH solution, smooth, thick wall, dextrinoid, apical with small pores, 1–2 µm. Basidia 17–30 (39) × 8–10 (13) µm, clavate, 4-sterigmate up to 3–4 µm long, 2–3 sterigmate occasional. Pleurocystidia (30) 33–59 (60) × (11) 12–21 (23) µm, utriform and lageniform, sparse, smooth, hyaline. Cheilocystidia 27–56 × (10) 11–20 (22) µm, utriform and lageniform, smooth, hyaline, crowded in hymenium. Lamellar edge fertile. Pileipellis a cutis, up to 100 µm thick, hyphae (35) 42–111 (148) × (6) 7–34 (35) µm, ovoid, subcylindrical, with brownish-yellow to dark brown pigment, thick wall, encrusting pigment on the outer hyphae. Veil hyphae (5) 6–30 (33) µm wide, present dark encrusting pigment, thick wall, colourless to light yellow, cylindrical and subcylindrical. Stipitipellis a cutis, hyphae (5) 6–22 (32) µm diam., ovoid and subcylindrical, pale brown, with encrusting pigment, thick wall. Clamp connections present in all tissues.

##### Habitat and distribution.

On humus of broad-leaved forest or coniferous and broad-leaved mixed forests in autumn.

##### Additional specimens examined.

China. Red Leaves Valley in Hanchongling, Dunhua City, Yanbian Korean Autonomous Prefecture, Jilin Province, 14 September 2019, Gu Rao (HMJAU 58783).

##### Notes.

*Coprinopsisjilinensis* is characterised by its small to medium-sized basidiomata, brown pileus with white hairs, smooth and dextrinoid basidiospores with small pores, pleurocystidia and cheilocystidia present. *C.jilinensis* forms a strongly-supported independent clade in ITS and LSU phylogeny trees (Figs 1, 2).

Morphologically and phylogenetic similar to *Coprinopsisjilinensis*, *C.uliginicola* is characterised by long basidiospores of 10–12(–15) µm, pleurocystidia absent and caulocystidia present, pileipellis no encrusting pigment (Smith 1972). Other similar species, *C.cineraria* is characterised by grey, hygrophanous and striate pileus, little short basidiospores (6.5–8.5 µm), pleurocystidia absent, pileipellis an epithelium (Takahashi 2000); *C.melanthina* is characterised by little long and subcolourless basidiospores (avl × avw = 10.5 × 5.8 µm), pleurocystidia absent (Kits van Waveren 1985); *C.pusilla* has small basidiomata, grey pileus, subcolourless and verrucose basidiospores (this study) and *Psathyrellasubagraria* has hygrophanous pileus, thick flesh and caulocystidia present (Smith 1972), both of which could be clearly distinguished from *C.jilinensis* in terms of morphology.

#### Coprinopsis pusilla

Taxon classificationFungiAgaricalesPsathyrellaceae

G. Rao, B. Zhang & Y. Li sp. nov.

E46DBB02-BC1F-50F0-B1E5-1481B0C21BBE

840298

**Figure 5.**
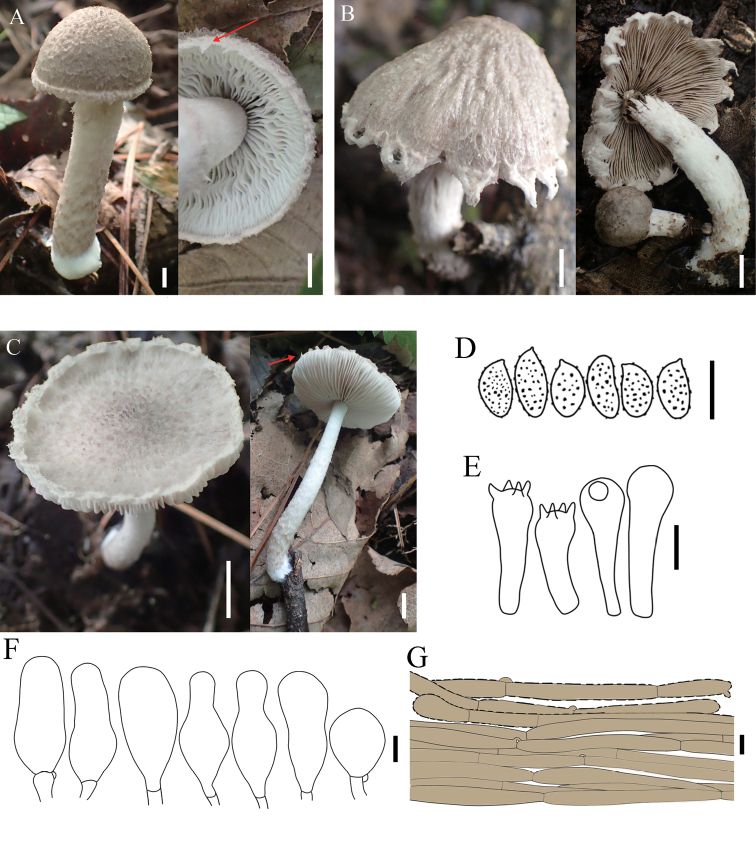
Basidiomata and microscopic features of *Coprinopsispusilla***A** collection HMJAU 58780 **B** collection HMJAU 58781 **C** collection HMJAU 58779 **D** basidiospores **E** basidia and basidioles **F** cheilocystidia **G** pileipellis. Scale bars: 5 mm (**A–C**); 10 µm (**D–G**).

**Figure 6.**
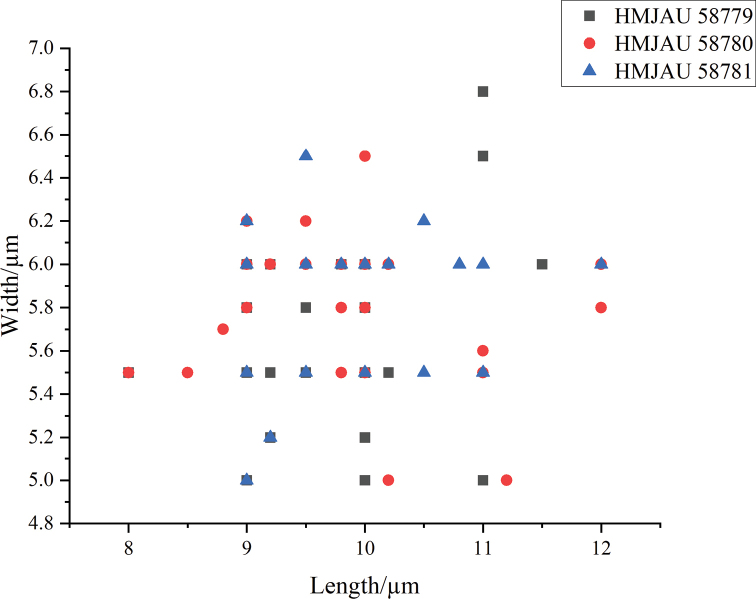
Scatter plot of basidiospores size in *Coprinopsispusilla*.

**Figure 7.**
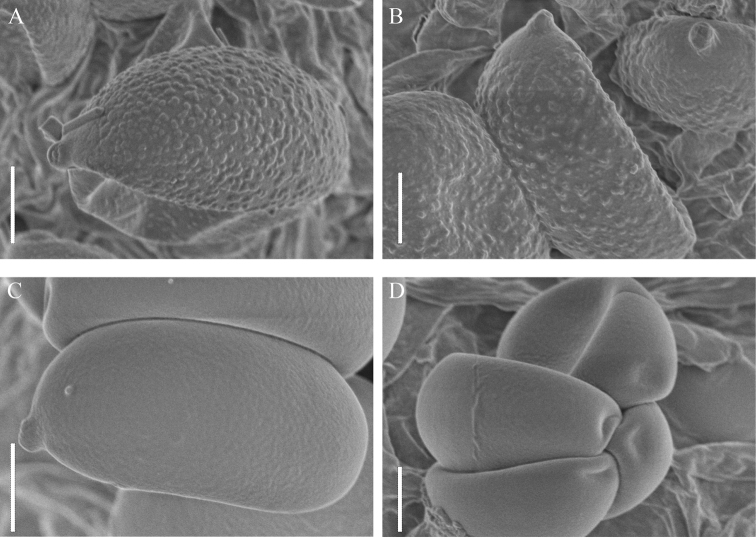
Scanning electron micrograph of basidiospores **A, B***Coprinopsispusilla***C, D***C.jilinensis*. Scale bars: 2 µm (**A–D**).

Figures 5 , 6 , 7A, B

##### Typification.

China. Red Leaves Valley in Hanchongling, Dunhua City, Yanbian Korean Autonomous Prefecture, Jilin Province, 21 August 2019, Gu Rao (HMJAU 58781 Holotype!).

##### Sequences ex holotype.

MZ398014 (ITS nrDNA), MZ398069 (LSU nrDNA).

##### Etymology.

The epithet “*pusilla*” refers to this species having small basidiomata.

##### Description.

Basidiomata very small to small. Pileus 21–29 mm broad, bell-shaped to hemispherical when young, then convex, flat to slightly reflexed at edges, with inconspicuous bulge at the middle, grey or greyish-white when dry, no record when wet, densely covered with flocculent hairs, sometimes central with blackish-grey squamous tapering to the edges, not slime, sometimes the edges crack, hygrophanous no record, veil remnants dense at edges, triangular, subtriangular or massive, not easily disappearing. Lamellae close or crowded, subwhite, greyish-white or coffee brown, flesh blond after drying, sinuate or adnexed, sometimes with vertical teeth, edges slightly toothed, concolorous, not deliquescent. Stipe 35–57 × 3–7 mm, cylindrical, subcylindrical, subequal or a little rough towards the base, white, cream white, hollow, a little fragile, not easy to detach from the cap, densely covered with white and flocculent hairs, brown, brownish-grey to brownish-yellow near the base, veil present at the stalk and cap joints, easily disappearing, no ring, the base with white mycelium. Spore print without record.

Basidiospores [90, 4, 3] 8–12 × 5–6.5 (6.8) µm, avl = 9.8 µm, avw = 5.8 µm, Q = 1.45–2.2 (2.24), Q_m_ = 1.70 ± 0.18 µm, oval, elliptic to long elliptic, subcolourless in 5% KOH and aqueous solution, surfaces verrucose, thin wall, no pores, not amyloid. Basidia (18) 19–32 (33) × 9–11 (12) µm, clavate, 4-sterigmate up to 3–4 µm long, 2-sterigmate occasional, without pseudoparaphyses. Pleurocystidia absent. Cheilocystidia (25) 27–53 (55) × (11) 13–21 µm, variable-shaped, subcylindrical, utriform, lageniform, reverse gourd-shaped and subcapitate, sphaeropedunculate elements present on gill edges, smooth, hyaline, thin wall to thick wall. Pileipellis a cutis, terminal hyphae (30) 31–84 (98) × (7) 8–17 (18) µm, with light brown pigment, mostly thick wall in the outer hyphae, present dark encrusting pigment, terminal hyphae present small cylindrical protrusions, about 3 × 3 μm. Veil hyphae (26) 27–100 (113) × (9) 10–19 (20) µm, without encrusting pigment, thick wall, colourless to yellowish, cylindrical, subcylindrical, clavate or irregular. Stipitipellis a cutis, hyphae (21) 22–87 (88) × (9) 10–19 (20) µm, encrusting pigment not observed, colourless to light yellow, cylindrical, subcylindrical, clavate or irregular, terminal hyphae present small cylindrical protrusions. Clamp connections present in all tissues.

##### Habitat and distribution.

On the dead and rotten wood of broad-leaved forest or coniferous and broad-leaved mixed forests in autumn.

##### Additional specimens examined.

China. Red Leaves Valley in Hanchongling, Dunhua City, Yanbian Korean Autonomous Prefecture, Jilin Province, 6 August 2019, G. Rao (HMJAU 58779, HMJAU 58780).

##### Notes.

*Coprinopsispusilla* has a variable macromorphology, but stable micromorphology, which is characterised by small basidiomata, greyish-white pileus, thick and distinct veil remnants at edges, subcolourless and verrucose basidiospores, no pore, the habitat on the decaying wood of broad-leaved trees. *C.pusilla* forms a strongly-supported independent clade in both ITS and LSU phylogeny trees (Figs 1, 2).

Morphologically and phylogenetically similar to *Coprinopsispusilla*, *C.melanthina* is characterised by larger brown pileus, fibrous veil at edges, longer basidiospores (avl = 10.5 µm) (Kits van Waveren 1985). *C.uliginicola* is characterised by large basidiomata, brown-black basidiospores, pore present (Smith 1972). *C.cineraria* is characterised by fibrous veil at the edges easily disappearing, smaller basidiospores (6.5–8.5 × 4–5 µm), pileipellis an epithelium (Takahashi 2000).

## Discussion

Here we report two new psathyrelloid species of *Coprinopsis* from northern China. There is no unified definition of “psathyrelloid”. Previously, “psathyrelloid” was mainly regarded as a species with the morphology of *Psathyrella*, including *Psathyrella* itself (Redhead et al. 2001). Örstadius et al. (2015) understood that “psathyrelloid” means that certain species of fungi with the morphology of *Psathyrella*, but belong to other genera in the phylogenetic analysis, so the species of *Psathyrella* were not included. These fungi were mainly distributed in *Coprinopsis* (Örstadius et al. 2015), *Typhrasa* (Örstadius et al. 2015; Wang et al. 2021), *Parasola* (Ganga and Manimohan 2019), *Homophron* (Örstadius et al. 2015), *Cystagaricus* (Örstadius et al. 2015), *Kauffmania* (Örstadius et al. 2015), *Lacrymaria* (Yan 2018) and so on. According to Redhead et al. (2001), coprinoid mushrooms were distributed in four genera: *Coprinus*, *Parasola*, *Coprinopsis* and *Coprinellus*. In Agaricaceae, Coprinoid mushrooms should also include *Montagnea*, *Podaxis* and *Xerocoprinus* (Moncalvo et al. 2002; Keirle et al. 2004). As there was no uniform standard, some fungi, such as *Parasolaconopilea* (Ganga and Manimohan 2019), were intermediate between psathyrelloid and coprinoid species. With the introduction of molecular systematics, the species of coprinoid and psathyrelloid intersected with each other. Örstadius et al. (2015) studied the morphology of many psathyrelloid species of Psathyrellaceae and believed that the lack of the typical pattern of pseudoparaphyses was their main morphological characteristic. Since psathyrelloid species belong to different genera, their distributions were not indicated phylogenetically in their study. Worldwide, there were, currently, at least fourteen psathyrelloid species of *Coprinopsis* (Picón 2010; Portugalete 2011; Örstadius et al. 2015; Crous 2017; Larsson and Örstadius 2017; Melzer et al. 2017), which belong to C.sect.Lanatulae in Schafe’s grouping system (Schafer 2010) and to three sections in Wächter’s grouping system, *Melanthinae* (including *C.cineraria*, *C.melanthina*, *C.uliginicola*, *C.jilinensis* and *C.pusilla*), *Canocipes* (including *C.aesontiensis*, *C.canoceps*, *C.lotinae*, *C.pannucioides*, *C.submicrospora* and *C.udicola*) and *Quartoconatae* (including *C.marcescibilis*, *C.musae* and *C.pseudomarcescibilis*). Currently, the known sequences of these species in these three sections are all the psathyrelloid species of *Coprinopsis*.

According to the results of BLASTn analyses, ITS showed higher interspecific variability, so the ITS sequence was more advantageous in reflecting the interspecific relationship of *Coprinopsis* than the LSU sequence. The sequence identity between *C.jilinensis* from Jilin Province, China and *C.uliginicola*MG712323 from Hubei Province, China, was 99.27%. Based on the molecular sequence alone, *C.uliginicola*MG712323 and *C.jilinensis* could be the same species and subsequent re-examination of this specimen is recommended.

In this study, some sequences of *Coprinopsis* were selected, which belong to C.sect.Melanthinae, C.sect.Canocipes and C.sect.Quartoconatae in the grouping system proposed by Wächter and Melzer (2020). In the phylogenetic analyses (Figs 1, 2), whether in the phylogenetic trees, based on ITS or LSU, the tree shape of C.sect.Melanthinae obtained was consistent and the branches were stable. However, C.sect.Canocipes and C.sect.Quartoconatae were somewhat different from the grouping described by Wächter and Melzer (2020), based on different sequences and analyses. In the phylogenetic trees, based on ITS, *C.udicola* and *C.submicrospora*, which originally belonged to C.sect.canocipes, formed a branch with three species belonging to C.sect.Quartoconatae in ML and BI trees. However, in the NJ tree, *C.udicola* formed a sister clade with three species belonging to C.sect.Quartoconatae. In the phylogenetic trees, based on LSU, C.sect.Canocipes and C.sect.Quartoconatae were well separated in the NJ tree, while in ML and BI trees, *C.musae* belonging to C.sect.Quartoconatae formed a sister clade with four species of C.sect.Canocipes. The branching relationships of phylogenetic trees, based on different molecular sequences and analyses may vary. Over-subdivision of sections would cause intersection. Subdivision of C.sect.Canocipes and C.sect.Quartoconatae may need to be reconsidered.

Coprinopsissect.Melanthinae has a relatively clear systematic differentiation (Wächter and Melzer 2020), which is consistent with the results of this study. Currently, there are only six species in this section, including the *C.lignicola* nom. prov. (GenBank no.: MG966286 and MF163181). Combined with the two newly-discovered species, the expression of this section is modified as follows:

**Coprinopsissect.Melanthinae Wächter & A. Melzer**

**Description.** Basidiomata very small to large, on humus or lignicolous. Pileus not radially sulcate, lamellae not deliquescent. Veil strongly developed, consisting of chains of subcylindrical, sometimes encrusted cells. Basidiospores medium to large-sized, ellipsoid to ovoid in side view, strikingly pale or brown, thin or thick-walled, germ pore absent or very indistinct, surfaces verrucose or smooth. Pseudoparaphyses absent. Basidia 4-sterigmate, 2–3-sterigmate occasional, always clavate, never polymorphic. Marginal cells of the lamellar edge predominantly utriform. Pleurocystidia present or absent. Cheilocystidia and clamps present.

*Coprinopsispusilla* has a variety of macroscopic morphology, including the shape and colour of the pileus, the shape of the veil and the thickness and length of the stipe and so on, which makes it difficult to determine whether it is the same species during the collection process, when the size of the basidiomata and ecological habits seem to be stable. The microscopic morphology of *C.pusilla* is relatively stable, including the size, shape, colour and decoration of basidiospores, the type of pileipellis and the presence or absence of cystidia. Interestingly, Coprinoid fungi are thought to be a taxon of dark-coloured basidiospores (Noordeloos et al. 2005), but *C.pusilla*, including *C.melanthina*, which is closely related to *C.pusilla*, have subcolourless basidiospores. Collection HMJAU 58779 and collection HMJAU 58780 were collected at approximately the same time in the same forest, which were not far apart from each other. However, due to the significant difference in macroscopic morphology, they were made into two collections. The macroscopic morphology of Collection HMJAU 58781, collected later, was also significantly different from the two specimens collected previously. Through the observation of the microscopic morphology of these collections and the phylogenetic analysis, combined with ITS and LSU molecular sequences, the results showed that the three collections were the same species.

Pleurocystidia is present in many species of *Coprinopsis*, such as *C.cinerea*, *C.jonesii* and *C.pseudoradiata* (Redhead et al. 2001), but in psathyrelloid species of *Coprinopsis*, species with pleurocystidia are rare. The pleurocystidia are sparsely distributed in the hymenium of *C.jilinensis*, but not present in *C.uliginicola*, which is closely related to *C.jilinensis*. In psathyrelloid species of *Coprinopsis*, only *C.pannucioides* (Larsson and Örstadius 2008), *C.udicola* (existing but rare) (Örstadius et al. 2015) and *C.jilinensis* have pleurocystidia.

*Psathyrellasubagraria* is a confusing species, described by Smith (1972) as it is very similar to *P.uliginicola* morphologically, with the main difference being that this species has pleurocystidia, which are mainly growing on humus. Since Smith (1972) introduced *P.subagraria*, no further reports have been made. Örstadius et al. (2015) moved *P.uliginicola* to *Coprinopsis*, based on molecular studies, the taxonomic status of *P.subagraria* being questionable. There are some differences between *P.subagraria* and *C.jilinensis* in macroscopic morphology, but they are similar in microscopic morphology. Smith (1972) described *P.subagraria* as having two basidiospores sizes [8–10 × 4–5 (10–12 × 4.5–5.5) µm], one of which was very close to the size of *C.jilinensis* [(8) 8.5–10 (10.2) × 4.5–5.9 (6) µm], requiring re-examination of the type specimens of *P.subagraria* in future studies.

### Key to fourteen psathyrelloid species of *Coprinopsis*

**Table:** 

1	Basidiospores no pore	**2**
–	Basidiospores with pore	**6**
2	Basidiospores distinctly pigmented	*** C. submicrospora ***
–	Basidiospores hyaline to very pale brown	**3**
3	Pileus < 20 mm, glabrous; pseudoparaphyses often present	*** C. musae ***
–	Pileus > 20 mm, hairy; pseudoparaphyses absent or unrecorded	**4**
4	Pileus hygrophanous, striate; basidiospores avl < 9 μm; pileipellis an epithelium	*** C. cineraria ***
–	Pileus not or scarcely hygrophanous, not striate; basidiospores avl > 9 μm; pileipellis a cutis	**5**
5	Pileus 25–80 mm, brown, fibrous veil at edges; basidiospores avl = 10.5 µm	*** C. melanthina ***
–	Pileus 21–29 mm, grey or greyish-white, veil dense at edges; basidiospores avl = 9.8 μm	*** C. pusilla ***
6	Basidiospores avl > 11.5 μm	**7**
–	Basidiospores avl < 11.5 μm	**10**
7	Pileus mostly brown, almost not striate, flocci at cap margin	**8**
–	Pileus mostly white or grey, striate, no flocci at cap margin	**9**
8	Basidiospores 13.3–14.5 µm; irregular flocci on cap margin	*** C. pseudomarcescibilis ***
–	Basidiospores 11.6–12.8 µm; denticulate flocci on cap margin	*** C. marcescibilis ***
9	Pseudoparaphyses present, pleurocystidia absent; caulocystidia present	*** C. lotinae ***
–	Pseudoparaphyses absent; pleurocystidia rare, close to gill edge; caulocystidia absent	*** C. udicola ***
10	Pleurocystidia present; cheilocystidia no long neck	**11**
–	Pleurocystidia absent; cheilocystidia part with long neck	**12**
11	Pileus yellowish-white, papillate-umbonate; caulocystidia present; tufty	*** C. pannucioides ***
–	Pileus brown, not papillate-umbonate; caulocystidia absent; solitary or scattered	*** C. jilinensis ***
12	Basidiomata medium-sized to large-sized (pileus > 50 mm); pileus pallid to greyish	*** C. uliginicola ***
–	Basidiomata very small to small (pileus < 50 mm); pileus not grey	**13**
13	Stipe < 60 mm long; basidia 4-sterigmate; cheilocystidia not inflated fusiform	*** C. canoceps ***
–	Stipe > 60 mm long; basidia 1-, 2-, 4-sterigmate; cheilocystidia part inflated fusiform	*** C. aesontiensis ***

## Supplementary Material

XML Treatment for Coprinopsis jilinensis

XML Treatment for Coprinopsis pusilla
